# Sexual Dimorphism in Adverse Pregnancy Outcomes - A Retrospective Australian Population Study 1981-2011

**DOI:** 10.1371/journal.pone.0158807

**Published:** 2016-07-11

**Authors:** Petra E. Verburg, Graeme Tucker, Wendy Scheil, Jan Jaap H. M. Erwich, Gus A. Dekker, Claire Trelford Roberts

**Affiliations:** 1 Robinson Research Institute, School of Medicine, University of Adelaide, Adelaide, South Australia, Australia; 2 Department of Obstetrics and Gynaecology, University Medical Center Groningen, University of Groningen, Groningen, The Netherlands; 3 Epidemiology Branch, SA Health, Adelaide, South Australia, Australia; 4 School of Medicine, University of Adelaide, Adelaide, South Australia, Australia; 5 Department of Obstetrics and Gynaecology, Lyell McEwin Hospital, Elizabeth Vale, South Australia, Australia; VU University Medical Center, NETHERLANDS

## Abstract

**Objectives:**

Sexual inequality starts *in utero*. The contribution of biological sex to the developmental origins of health and disease is increasingly recognized. The aim of this study was to assess and interpret sexual dimorphisms for three major adverse pregnancy outcomes which affect the health of the neonate, child and potentially adult.

**Methods:**

Retrospective population-based study of 574,358 South Australian singleton live births during 1981–2011. The incidence of three major adverse pregnancy outcomes [preterm birth (PTB), pregnancy induced hypertensive disorders (PIHD) and gestational diabetes mellitus (GDM)] in relation to fetal sex was compared according to traditional and fetus-at-risk (FAR) approaches.

**Results:**

The traditional approach showed male predominance for PTB [20–24 weeks: Relative Risk (RR) M/F 1.351, 95%-CI 1.274–1.445], spontaneous PTB [25–29 weeks: RR M/F 1.118, 95%-CI 1.044–1.197%], GDM [RR M/F 1.042, 95%-CI 1.011–1.074], overall PIHD [RR M/F 1.053, 95%-CI 1.034–1.072] and PIHD with term birth [RR M/F 1.074, 95%-CI 1.044–1.105]. The FAR approach showed that males were at increased risk for PTB [20–24 weeks: RR M/F 1.273, 95%-CI 1.087–1.490], for spontaneous PTB [25–29 weeks: RR M/F 1.269, 95%-CI 1.143–1.410] and PIHD with term birth [RR M/F 1.074, 95%-CI 1.044–1.105%]. The traditional approach demonstrated female predominance for iatrogenic PTB [25–29 weeks: RR M/F 0.857, 95%-CI 0.780–0.941] and PIHD associated with PTB [25–29 weeks: RR M/F 0.686, 95%-CI 0.581–0.811]. The FAR approach showed that females were at increased risk for PIHD with PTB [25–29 weeks: RR M/F 0.779, 95%-CI 0.648–0.937].

**Conclusions:**

This study confirms the presence of sexual dimorphisms and presents a coherent framework based on two analytical approaches to assess and interpret the sexual dimorphisms for major adverse pregnancy outcomes. The mechanisms by which these occur remain elusive, but sex differences in placental gene expression and function are likely to play a key role. Further research on sex differences in placental function and maternal adaptation to pregnancy is required to delineate the causal molecular mechanisms in sex-specific pregnancy outcome. Identifying these mechanisms may inform fetal sex specific tailored antenatal and neonatal care.

## Introduction

The foundation for the health of children and both women and men is established during intrauterine life when the fetus is said to be programmed by the intrauterine environment. The “developmental origins of health and disease” hypothesis indicates long-term health consequences for individuals with a low birth weight[1].

Adverse pregnancy outcomes, such as preterm birth (PTB), pregnancy induced hypertensive disorders (PIHD) and gestational diabetes mellitus (GDM) do not only have an immense influence on the mother, but also on the baby. Pregnancy complications are associated with impaired development of the fetus, neonate and infant. Both women who had preeclampsia and the babies born to them are at increased risk for later adult onset diseases such as hypertension, cardiovascular disease and type 2 diabetes[2]. Preterm born babies are more likely to die or suffer significant long-term health problems including cerebral palsy, vision impairment and lung disease[3]. Offspring from mothers with GDM are at increased risk of developing obesity, impaired glucose tolerance, Type 2 Diabetes and cardio-vascular disease in adulthood[4].

The National Institutes of Health (NIH) recently highlighted the importance of evaluating the sex differences in health and disease. This forms one of the main goals of the NIH strategic plan ‘Moving into the Future with New Dimensions and Strategies for Women’s Health Research: A Vision for Women’s Health Research’ (http://orwh.od.nih.gov/research/priorities.asp).

Adverse pregnancy outcomes are heterogeneous conditions and their pathophysiology is not fully understood. During the last few decades interest has grown in identifying risk factors for adverse pregnancy outcome to help understand the underlying mechanisms and potentially prevent them in the future. Several themes have emerged, including the importance of the placenta and the presence of sexual dimorphism in progression and development of child and adult diseases.

It has been suggested that male fetal sex is an independent risk factor for adverse pregnancy outcome and that female fetuses have an advantage over male fetuses[5]. Several studies have found an association between male fetal sex and excess perinatal mortality and morbidity[6–13]. Women carrying a male fetus appear to be at an increased risk for PIHD[7,8,10,13], PTB[6–8,10,12–15] and GDM[10,13,15].

However, the literature is not consistent. Although spontaneous PTB is more prevalent in male fetuses, iatrogenic PTB is more prevalent in female foetuses[8,11,12]. Also, some studies suggest that preeclampsia complicated with PTB is more prevalent in female foetuses[7–10]. And, some recent studies found no sexual dimorphism for overall PIHD[10,12,13,15], for PIHD complicated by PTB[13], nor for GDM[12].

Incidence, prevalence, pathophysiology and health outcomes for a number of common diseases are different between the sexes. Sex inequality starts *in utero* and the contribution of biological sex to the”developmental origins of health” and disease is increasingly recognized[1].

In neonatal and pediatric care, studies have shown sex specific differences in the response to maternal conditions, such as asthma[16] and to antenatal glucocorticoid treatment for women with threatened preterm birth[17,18].

Also, sex differences in lung development, disease course and response to treatment have been well documented[19]. These differences are present as early as 16–24 weeks of gestation. Females have a lower number of bronchioles compared with males, but females mature faster. Also, surfactant is produced earlier in gestation by females compared to males. In neonates, females have higher expiratory flow rates corrected for size compared to males and this difference remains present throughout the life span[19]. Childhood lung conditions, such as asthma, atopy and allergic rhinitis are more common in boys versus girls[20]. A number of intrinsic and environmental risk factors for asthma are known, but early life events, such as preterm birth, may contribute.

Traditionally, population studies have determined the incidence and therefore risk of a perinatal outcome, as the number of cases in a certain gestation group divided by the number of births at that gestation[7,21] or divided by all births in the population[6,8–10,12,13,15]. Other studies have used variations on this approach[11,22,23].

However, the incidence of any pregnancy related event at any gestation is defined as the number of new cases of the event that occurs within that gestational week divided by the number of candidates at risk for the event at that gestation[24].Therefore, the so-called “fetuses-at-risk” (FAR) approach has been suggested for use in perinatal research[24,25]. This approach identifies fetuses as the candidates at risk for perinatal events rather than neonates. Similar to a survival analysis it takes into account the remaining fetuses at risk such that as gestation proceeds and babies are born preterm, only outcomes for the remaining fetuses are analysed. Therefore, it is thought that the FAR approach may provide more insight into the causal links between length of gestation and perinatal outcomes. The FAR approach is recognized and accepted in the literature for stillbirth, but to our knowledge this approach has not been used in studies on sexual dimorphism in perinatal outcome before[24].

By using a well-curated Australian database, this study aims to present both traditional and FAR approaches to analysis providing a coherent framework to assess and interpret the sexual dimorphism for three major adverse perinatal outcomes.

## Materials and Methods

### Study population

This retrospective population-based cohort study included all singleton live births in South Australia (SA), Australia, between January 1981 and December 2011, recorded in the South Australian Perinatal Statistics Collection (SAPSC) maintained by the Pregnancy Outcome Unit (POU) of SA Health. The SAPSC collects information regarding the characteristics and outcome of all births with a gestation of 20–42 weeks and a birth weight ≥ 400 grams in South Australia, notified by hospital and homebirth midwives and neonatal nurses using a standardised Supplementary Birth Record (SBR).

Gestational age was based on dating ultrasound (performed at 8–13 weeks gestation) supported by the first day of the last menstrual period or by review of other ultrasonography and reported as completed weeks.

### Study Outcomes

The studied maternal variables were age, parity, gravidity and ethnicity. The neonatal variables included infant sex, birth weight, gestational age at birth, and pregnancy outcome, defined as PTB, PIHD and GDM.

Analyses were performed on six gestational age categories, as defined in a previous large Norwegian population study[7] with four PTB categories (20–24, 25–29, 30–33 and 34–36 weeks), and two term categories (37–39 and 40–42 weeks). Spontaneous birth was defined as birth without any pharmacological, surgical or other intervention undertaken to stimulate the onset of labour. Iatrogenic preterm birth was defined as preterm induction of birth and/or caesarean section due to other pregnancy morbidities, mainly preeclampsia or intrauterine growth restriction (IUGR).

PIHD was defined as blood pressure ≥ 140/90 on two occasions at least four hours apart, or ≥ 170/110 on one occasion, ± proteinuria. GDM was listed in the SAPSC when the clinician documented that the woman had GDM based on the criteria of the hospital or laboratory where the test was performed. Routine glucose challenge testing for GDM was introduced in SA in the early 1980’s. GDM defined as a fasting glucose ≥ 5.5. mmol/L and /or 2hr value ≥ 8 mmol/L was uniformly reported from 1987. For the analyses of GDM, all births recorded between 1987 and 2011 were used.

### Statistical Analyses

Data analysis was performed with SPSS version 21.0 (SPSS inc., 2013). The Student t-test was used to compare continuous variables, and χ^2^-test was used for categorical variables. Differences were considered significant when the *p*-value was less than 0.05.

**Fig 1:**
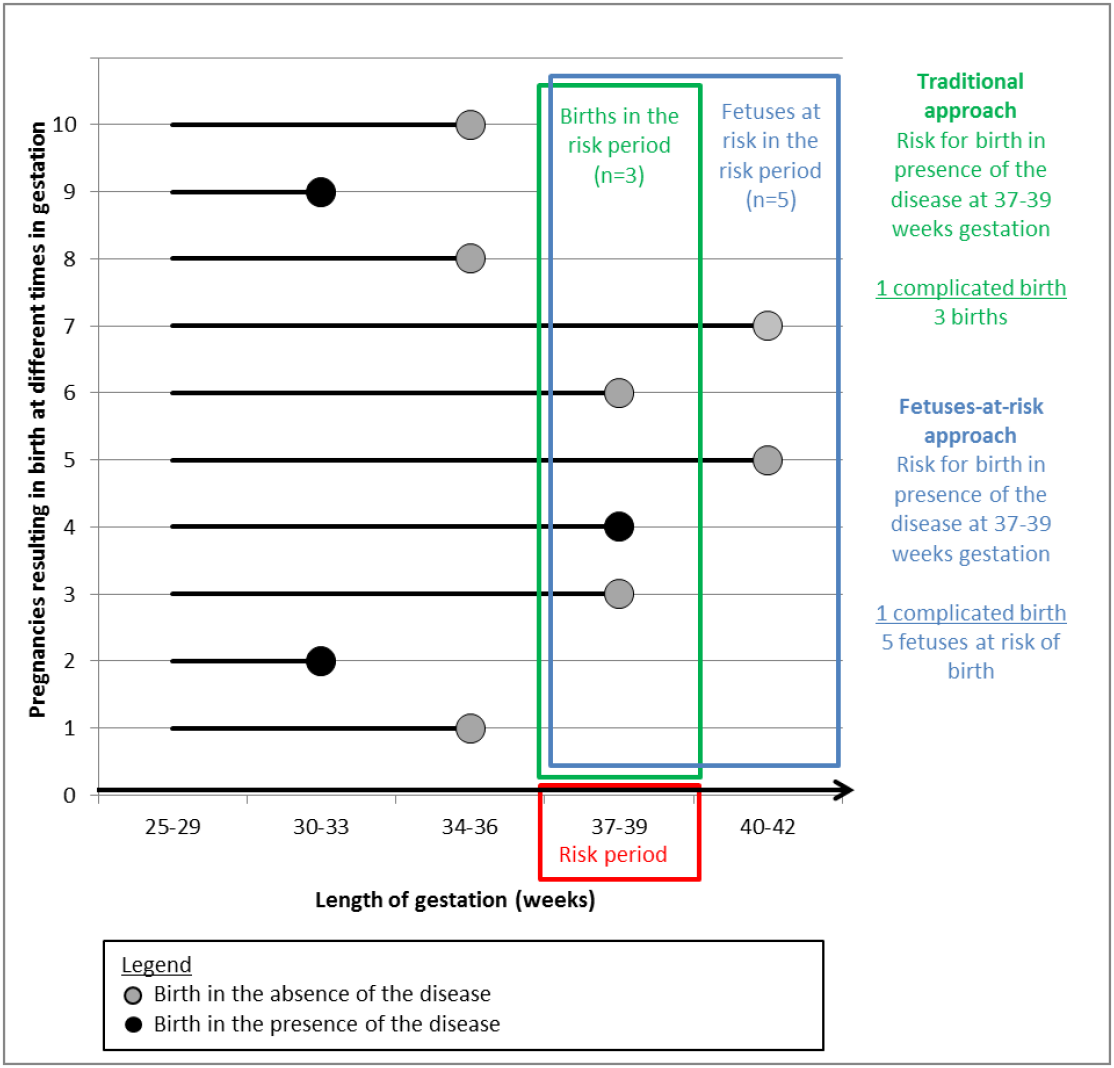
Schematic depiction of pregnancy course and options for calculating the gestational age-specific disease rate in 10 hypothetical pregnancies. *Traditional approach*: Number of affected births in a gestational group divided by the number of total births within that gestation group = 1/3. *Fetuses at risk approach*: Number of affected births at gestational group divided by the number of fetuses at risk for a disease at that gestation group = 1/5. Modified after Joseph et al(24).

Sexual dimorphisms in adverse pregnancy outcomes were calculated according to two approaches as defined by Joseph[24] (Fig 1). The traditional approach employs the incidence of births with the adverse pregnancy outcome divided by the number of births at that gestation. The FAR approach employs the incidence of births with the adverse pregnancy outcome divided by the number of fetuses at risk of birth at that gestation. Fetal sex ratios in both approaches were calculated as relative risks for males versus females (RR M/F). 95% Confidence Intervals (95%-CI) were tabulated to demonstrate variability of the estimate.

### Ethical approval

The Human Research Ethics Committee (HREC) of the South Australian Department of Health [HREC/13/SAH/97] approved the study protocol. The existence of personal identifying information in the SAPSC was eliminated to ensure that confidentially of all patient records was maintained.

## Results

Of a total of 596,600 births recorded in the SAPSC during the study period, 574,358 (96.3%) births were eligible for the study, including 295,724 (51.5%) male and 278,634 (48.5%) female neonates. The overall M/F-ratio was 1.06. The demographics and obstetric characteristics are presented in Table 1. There were no significant differences between the groups regarding maternal age, parity, gravidity, and ethnicity. Overall, male fetuses had a lower mean gestational age at birth (p <0.001) and a higher mean birth weight (p <0.001).

**Table 1:** Demographics and obstetric characteristics by fetal sex.

	Males		Females	
Characteristic	295,724	(51.5)	278,634	(48.5)
Maternal age (years)	28.6	± 5.5	28.6	± 5.5
Parity	0.96	± 1.1	0.96	± 1.1
Gravidity	1.47	± 1.6	1.47	± 1.6
Gestational age at birth (weeks)	39.14	± 1.9	39.18	± 1.8
Birth weight (g)	3,452	± 565	3,324	± 534
Ethnicity*				
- Caucasian	271,663	(51.5)	255,826	(48.5)
- Aboriginal and/or Torres Strait Islanders	7,566	(50.9)	7,301	(49.1)
- Asian	13,094	(51.7)	12,210	(48.3)
- Other	3,391	(50.8)	3,289	(49.2)

Values are presented as mean ± SD or *n* (%).

* Analysis of 574,340 births [295,714 (51.5%) males versus 278,626 (48.5%) females].

### Preterm birth

**Fig 2:**
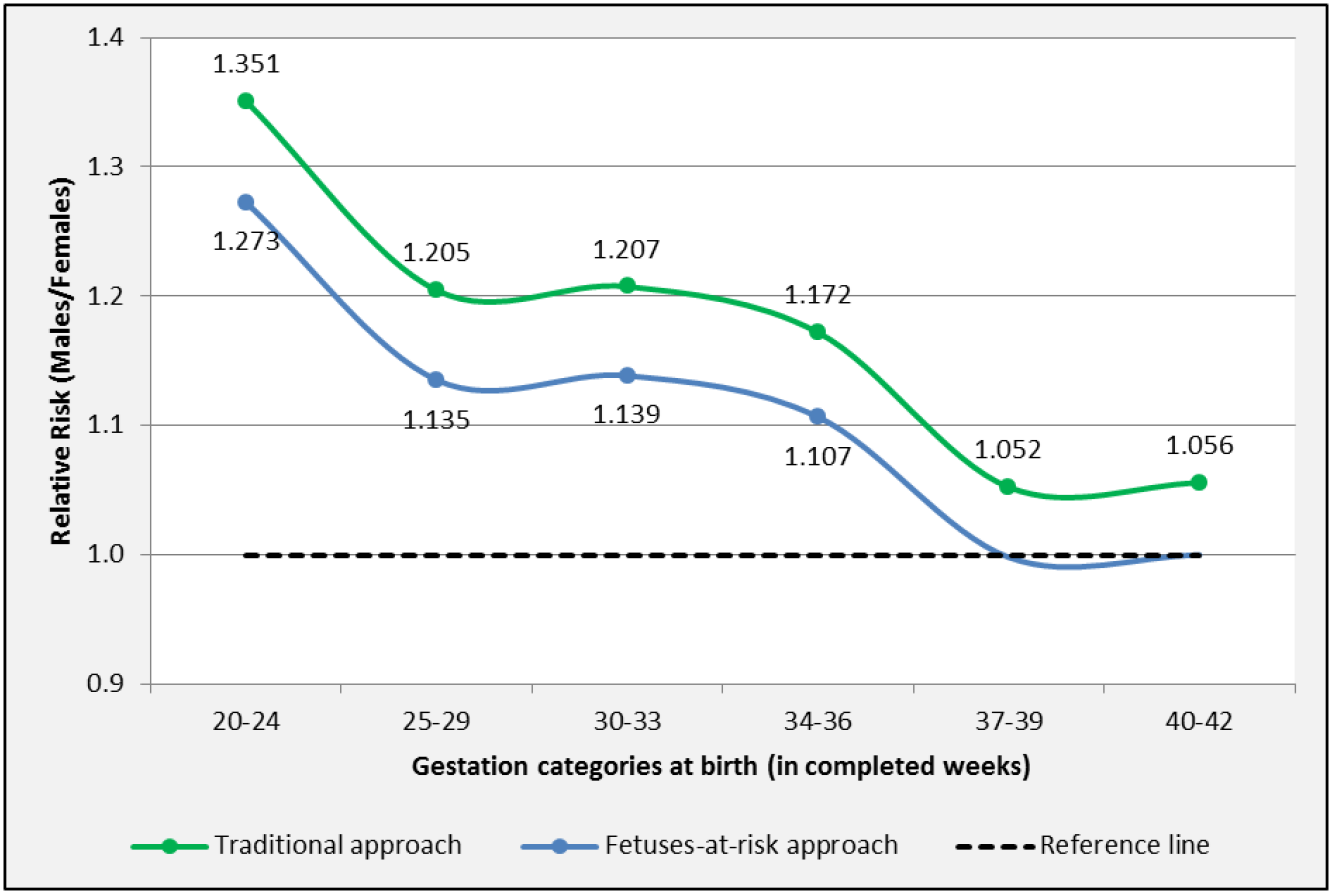
Sexual dimorphism for length of gestation in categories. Births in South Australia 1981–2011. Marked points represent significant RR M/F.

The sexual dimorphism for length of gestation is shown in Table 2 and Fig 2. The traditional approach showed a male predominance for PTB [RR M/F 1.351, 95%-CI 1.274–1.445 (20–24 weeks)] and term birth [RR 1.052, 95%-CI 1.051–1.054 (37–39 weeks)]. The FAR approach showed an increased risk of PTB in women carrying a male fetus [RR M/F 1.273, 95%-CI 1.087–1.490 (20–24 weeks)]. At term unity in RR M/F was reached.

**Table 2:** Sexual dimorphism for length of gestation in categories.

Traditional approach							
	Total births	Males (n)		Females (n)			
Gestation (weeks)		Total		Total		RR M/F*	95%-CI**
20–24	630	362		268		**1.351**	1.274–1.445
25–29	2,436	1,331		1,105		**1.205**	1.176–1.236
30–33	6,011	3,288		2,723		**1.207**	1.189–1.227
34–36	23,891	12,891		11,000		**1.172**	1.164–1.180
37–39	223,703	114,701		109,002		**1.052**	1.051–1.054
40–42	317,687	163,151		154,536		**1.056**	1.055–1.057
Fetuses-at-risk approach							
		Males (n)		Females (n)			
Gestation (weeks)		Total	FAR***	Total	FAR***	RR M/F*	95%-CI**
20–24		362	295,724	268	278,634	**1.273**	1.087–1.490
25–29		1,331	295,362	1,105	278,366	**1.135**	1.048–1.229
30–33		3,288	294,031	2,723	277,261	**1.139**	1.083–1.198
34–36		12,891	290,743	11,000	274,538	**1.107**	1.079–1.134
37–39		114,701	277,852	109,002	263,538	0.998	0.992–1.004
40–42		163,151	163,151	154,536	154,536	**1.000**	1.000–1.000

Births in South Australia 1981–2011.

* RR M/F denotes the relative risk for the proportion male fetuses versus female fetuses.

** CI denotes Confidence Interval. Results of univariate analyses. Bold values indicate statistical significance.

*** FAR denotes Fetuses-at-risk

#### Spontaneous preterm birth

**Fig 3:**
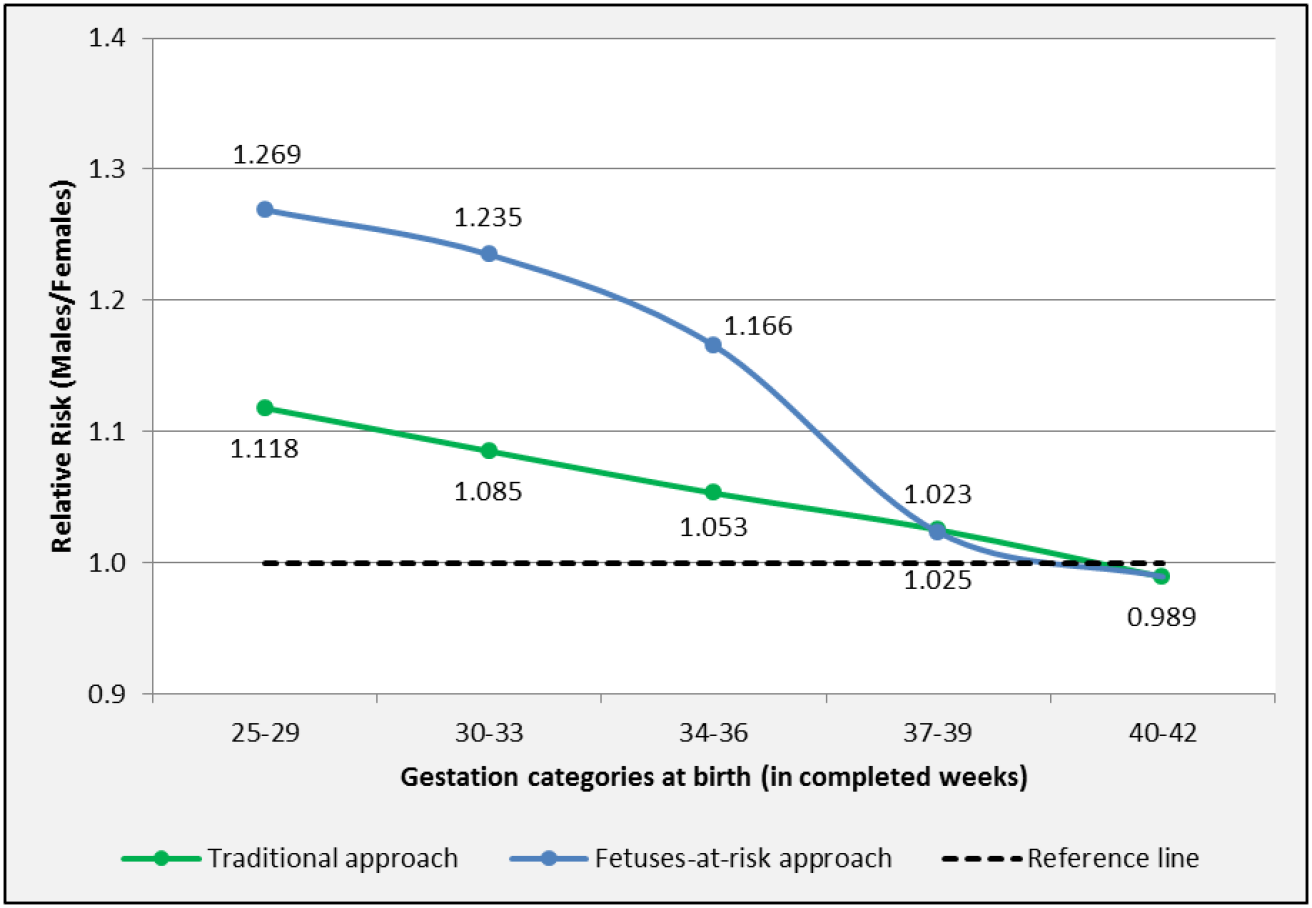
Sexual dimorphism for spontaneous birth by length of gestation. Births in South Australia 1981–2011. Marked points represent significant RR M/F.

The traditional approach showed a male predominance for spontaneous PTB [RR M/F 1.118, 95%-CI 1.044–1.197 (25–29 weeks)] (Table 3 and Fig 3). At term, a “cross-over” was observed. In the gestation category 37–39 weeks spontaneous birth showed a male predominance (RR M/F 1.025, 95%-CI 1.018–1.033), whereas spontaneous birth was more prevalent in female bearing pregnancies between 40–42 weeks gestation (RR M/F 0.989, 95%-CI 0.985–0.994).

**Table 3:** Sexual dimorphism for spontaneous birth by length of gestation in categories.

Traditional approach						
	Males (n)		Females (n)			
Gestation (weeks)	Spontaneous birth	All birth	Spontaneous birth	All birth	RR M/F*	95%-CI**
25–29	816	1,331	606	1,105	**1.118**	1.044–1.197
30–33	1,950	3,288	1,489	2,723	**1.085**	1.037–1.134
34–36	8,316	12,891	6,737	11,000	**1.053**	1.033–1.074
37–39	64,984	114,701	60,245	109,002	**1.025**	1.018–1.033
40–42	108,920	163,151	104,282	154,536	**0.989**	0.985–0.994
Fetuses-at-risk approach						
	Males (n)		Females (n)			
Gestation (weeks)	Spontaneous birth	FAR***	Spontaneous birth	FAR***	RR M/F*	95%-CI**
25–29	816	295,362	606	278,366	**1.269**	1.143–1.410
30–33	1,950	294,031	1,489	277,261	**1.235**	1.155–1.321
34–36	8,316	290,743	6,737	274,538	**1.166**	1.129–1.203
37–39	64,984	277,852	60,245	263,538	**1.023**	1.013–1.033
40–42	108,920	163,151	104,282	154,536	**0.989**	0.985–0.994

Births in South Australia 1981–2011.

* RR M/F denotes the relative risk for the proportion male fetuses versus female fetuses.

** CI denotes Confidence Interval. Results of univariate analyses. Bold values indicate statistical significance.

*** FAR denotes Fetuses-at-risk

Using the FAR approach, males were at increased risk for spontaneous PTB [RR M/F 1.269, 95%-CI 1.143–1.410 (25–29 weeks)]. At term a “cross-over” was observed and male fetuses were at increased risk for spontaneous birth between 37–39 weeks (RR M/F 1.023, 95%-CI 1.013–1.033), whereas female fetuses were at increased at risk for spontaneous birth between 40–42 weeks (RR M/F 0.989, 95%-CI 0.985–0.994).

#### Iatrogenic preterm birth

**Fig 4:**
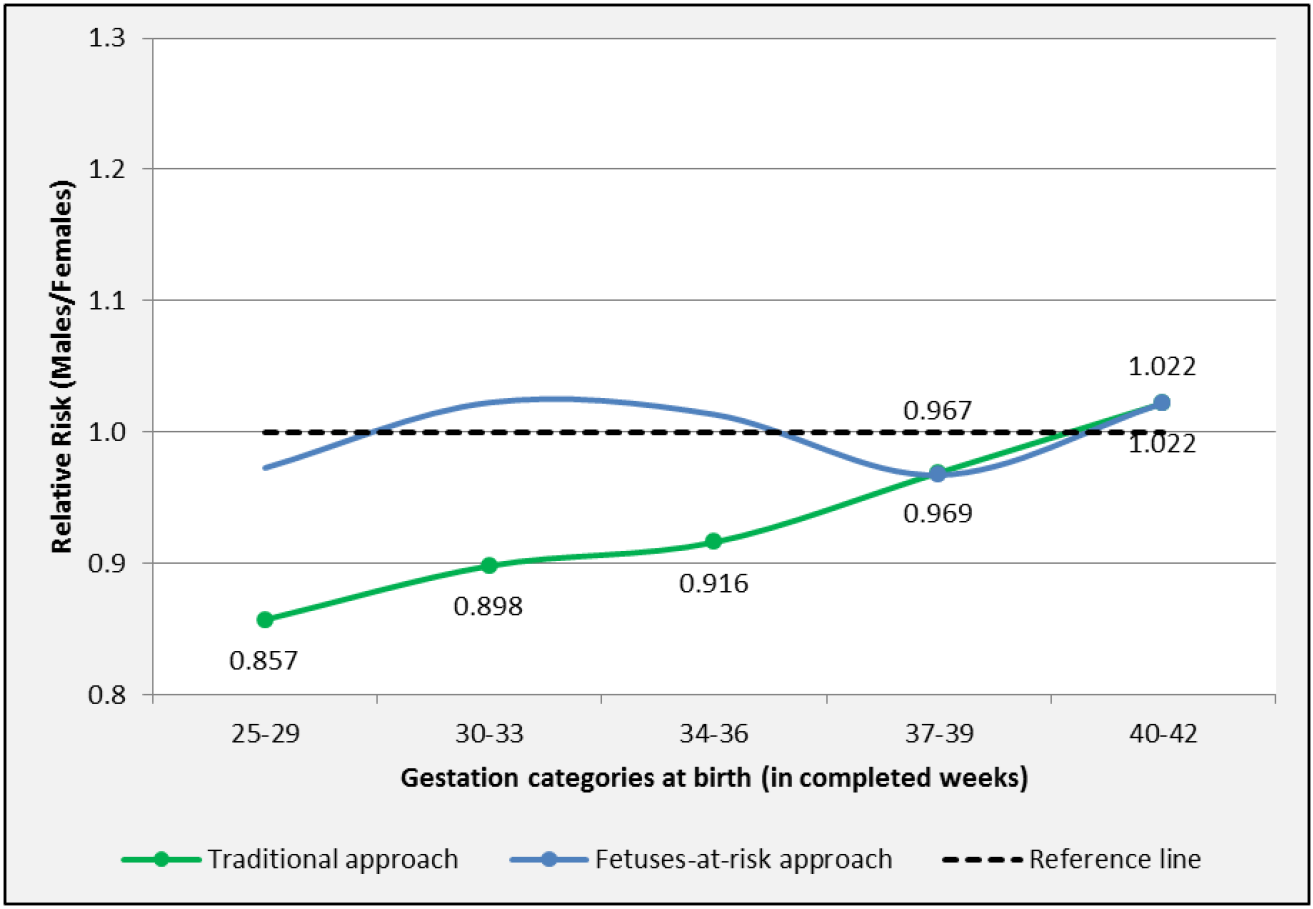
Sexual dimorphism for iatrogenic birth by length of gestation. Births in South Australia 1981–2011. Marked points represent significant RR M/F.

The traditional approach observed a female predominance for iatrogenic PTB [RR M/F 0.857, 95%-CI 0.780–0.941 (25–29 weeks)] (Table 4 and Fig 4). At term, a “cross-over” was observed. In the gestation category 37–39 weeks iatrogenic birth showed a female predominance (RR M/F 0.969, 95%-CI 0.960–0.978), whereas in the 40–42 gestation group, iatrogenic birth was more prevalent in male bearing pregnancies (RR M/F 1.022, 95%-CI 1.012–1.032).

**Table 4:** Sexual dimorphism for iatrogenic birth by length of gestation in categories.

Traditional approach						
	Males (n)		Females (n)			
Gestation (weeks)	Iatrogenic birth	All birth	Iatrogenic birth	All birth	RR M/F*	95%-CI**
25–29	515	1,331	499	1,105	**0.857**	0.780–0.941
30–33	1,338	3,288	1,234	2,723	**0.898**	0.847–0.952
34–36	4,575	12,891	4,263	11,000	**0.916**	0.886–0.947
37–39	49,717	114,701	48,757	109,002	**0.969**	0.960–0.978
40–42	54,231	163,151	50,254	154,536	**1.022**	1.012–1.032
Fetuses-at-risk approach						
	Males (n)		Females (n)			
Gestation (weeks)	Iatrogenic birth	FAR***	Iatrogenic birth	FAR***	RR M/F*	95%-CI**
25–29	515	295,362	499	278,366	0.973	0.860–1.100
30–33	1,338	294,031	1,234	277,261	1.022	0.946–1.104
34–36	4,575	290,743	4,263	274,538	1.013	0.972–1.056
37–39	49,717	277,852	48,757	263,538	**0.967**	0.956–0.978
40–42	54,231	163,151	50,254	154,536	**1.022**	1.012–1.032

Births in South Australia 1981–2011.

* RR M/F denotes the relative risk for the proportion male fetuses versus female fetuses.

** CI denotes Confidence Interval. Results of univariate analyses. Bold values indicate statistical significance.

*** FAR denotes Fetuses-at-risk

The FAR approach showed that iatrogenic PTB had a pattern close to unity. At term, a ‘cross-over’ was observed. Females were at increased risk for iatrogenic birth at 37–39 weeks gestation [RR M/F 0.967, 95%-CI 0.956–0.978], whereas males were at increased risk for iatrogenic birth at 40–42 weeks [RR M/F 1.022, 95%-CI 1.012–1.032].

### Pregnancy Induced Hypertensive Disorders and Gestational Diabetes Mellitus

The rates for hypertensive disorders and diabetes are shown in Table 5. There were no significant differences between the groups regarding pre-gestational diabetes and chronic hypertension. GDM showed a male predominance (RR M/F 1.042, 95%-CI 1.011–1.074) and overall PIHD showed a male predominance (RR M/F 1.053, 95%-CI 1.034–1.072).

**Table 5:** Diabetes and hypertensive disorders according to fetal sex.

	Males		Females		RR M/F*	95%-CI**
	295,724	(51.5)	278,634	(48.5)		
Hypertensive disorders						
Chronic hypertension	3,101	(1.0)	2,835	(1.0)	1.031	0.980–1.084
PIHD	22,966	(7.8)	20,550	(7.4)	**1.053**	**1.034–1.072**
Diabetes***						
Pre-gestational diabetes	1,083	(0.5)	992	(0.4)	1.028	0.943–1.120
GDM	8,630	(3.6)	7,796	(3.5)	**1.042**	**1.011–1.074**

Births in South Australia 1981–2011. Values are presented as *n* (%).

* RR M/F denotes the relative risk for the proportion male fetuses versus female fetuses.

** CI denotes Confidence Interval. Results of univariate analyses. Bold values indicate statistical significance.

***Analysis of 460,749 subjects [237,333 males (51.5%) vs. 223,416 females (48.5%)]

**Fig 5:**
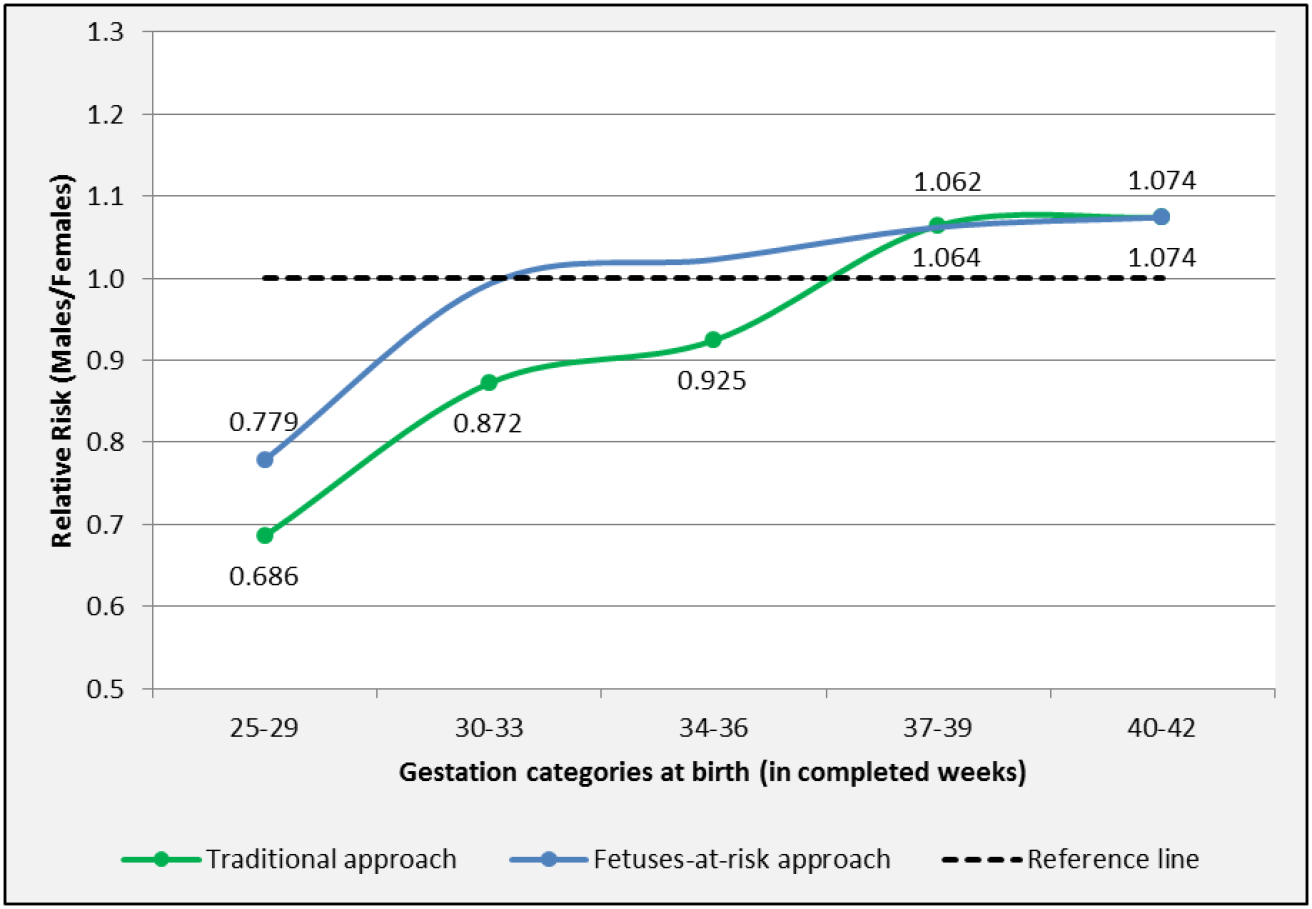
Sexual dimorphism for PIHD by length of gestation in categories. Births in South Australia 1981–2011. Marked points represent significant RR M/F.

In the traditional approach, PIHD associated with PTB showed a female predominance [RR M/F 0.686, 95%-CI 0.581–0.811 (25–29 weeks)], while PIHD associated with term birth was more prevalent in males [RR M/F 1.074, 95%-CI 1.044–1.105 (40–42 weeks)] (Table 6 and Fig 5). Using the FAR approach, female fetuses were at increased risk for PIHD associated with very PTB [RR M/F 0.779, 95%-CI 0.648–0.937 (25–29 weeks)]. Male fetuses were at increased risk for PIHD associated with term birth [1.074, 95%-CI 1.044–1.105 (40–42 weeks)].

**Table 6:** Sexual dimorphism for PIHD by length of gestation in categories.

Traditional approach						
	Males (n)		Females (n)			
Gestation (weeks)	PIHD births	All births	PIHD births	All births	RR M/F*	95%-CI**
25–29	205	1,331	248	1,105	**0.686**	**0.581–0.811**
30–33	592	3,288	562	2,723	**0.872**	**0.786–0.968**
34–36	1,844	12,891	1,702	11,000	**0.925**	**0.870–0.982**
37–39	10,703	114,701	9,556	109,002	**1.064**	**1.037–1.093**
40–42	9,610	163,151	8,472	154,536	**1.074**	**1.044–1.105**
Fetuses-at-risk approach						
	Males (n)		Females (n)			
Gestation (weeks)	PIHD births	FAR***	PIHD births	FAR***	RR M/F*	95%-CI**
25–29	205	295,362	248	278,366	**0.779**	**0.648–0.937**
30–33	592	294,031	562	277,261	0.993	0.885–1.115
34–36	1,844	290,743	1,702	274,538	1.023	0.958–1.092
37–39	10,703	277,852	9,556	263,538	**1.062**	**1.034–1.091**
40–42	9,610	163,151	8,472	154,536	**1.074**	**1.044–1.105**

Births in South Australia 1981–2011. Values are presented as *n* (%).

* RR M/F denotes the relative risk for the proportion male fetuses versus female fetuses.

** CI denotes Confidence Interval. Results of univariate analyses. Bold values indicate statistical significance.

*** FAR denotes Fetuses-at-risk

## Discussion

This large 30-year South Australian population study confirms the presence of clear sexual dimorphisms for PTB, PIHD and GDM.

### Sexual dimorphisms

#### Preterm birth

Both the traditional and the FAR approaches demonstrated a male predominance for all PTB. The traditional approach showed that, of all births at 20–24 weeks gestation, PTB was 35.1% more common in male-bearing pregnancies. The FAR approach showed that, of all ongoing pregnancies at 20–24 weeks gestation, the male-bearing pregnancies were at 27.3% increased risk for PTB.

Both the traditional and the FAR-approaches also showed a male predominance for spontaneous PTB. The traditional approach showed that of all births at 25–29 weeks gestation, spontaneous PTB was 11.8% more common in male-bearing pregnancies. The FAR approach showed that of all ongoing pregnancies at 25–29 weeks gestation the male-bearing pregnancies were at 26.9% increased risk of a spontaneous PTB at 25–29 weeks gestation. The pattern for iatrogenic PTB was not as consistent between approaches. The traditional approach showed that of all births at 25–29 weeks gestation, iatrogenic PTB was 14.3% more common in female-bearing pregnancies. In contrast, the FAR approach showed that of all ongoing pregnancies at 25–29 weeks gestation the risk of iatrogenic PTB at 25–29 weeks gestation was equal in both sexes.

The male predominance for spontaneous PTB was observed in previous relatively small studies[8,11,12] and the female predominance for iatrogenic PTB was observed in two of these studies[8,11].

The observation that using the FAR approach the risk for iatrogenic PTB was equal for both sexes at this gestation suggests that the susceptible male fetuses were lost earlier in pregnancy and therefore were lost to the analysis in subsequent gestation groups, hence elevating the proportion of females remaining at this stage of gestation.

A higher male/female ratio for PTB was previously described in both large population studies[6,7,9,21], and in relatively smaller studies[8,10–13]. A pronounced male predominance has also been documented in pregnancy losses occurring before 20 weeks gestation where the overall sex-ratio was 1.25, with the highest male excess in first trimester losses (sex-ratio 2.00–2.50)[22]. Interestingly, in this particular study, in the first trimester, fetuses with normal chromosomal morphology showed a sex-ratio of 1.50–1.80[22]. We observed that fetuses were at greatest risk for birth, prior to the age of viability (<24 weeks) if they were male. This was of a similar magnitude reported in the study by Byrne and Warburton[22].

#### Pregnancy Induced Hypertensive Disorders

Overall PIHD showed a male predominance. Previous studies have shown conflicting results. The majority of previous studies documented no sexual dimorphism for overall PIHD[10,12,13,15], whereas others documented a female predominance[23] or similar results to our study[7,9]. Some[7–10], but not all, studies[12,13,15] distinguished between PIHD resulting in PTB and PIHD resulting in term birth. The reason the female predominance disappears in the overall PIHD group, is most likely because the vast majority of PIHD is at term.

Both traditional and FAR approaches demonstrated that female fetuses showed a higher incidence of PIHD associated with PTB. The traditional approach showed that of all births at 25–29 weeks gestation, pregnancies complicated by PIHD were 31.4% more common in female-bearing pregnancies. The FAR approach showed that of all ongoing pregnancies at 25–29 weeks gestation, female-bearing pregnancies were at 22.1% increased risk of PIHD. In addition, male fetuses showed a higher prevalence and an increased risk for PIHD with term birth. Interestingly, the “cross-over” from female to male predominance in both approaches was different, suggesting that fetal sex may not be a risk factor for PIHD resulting in birth between 30 and 36 weeks of gestation.

#### Gestational Diabetes Mellitus

Women were at increased risk for GDM when carrying a male fetus. Previous studies have shown conflicting results. A relatively small study found no sexual dimorphism for GDM[12], while another study showed a greater maternal insulin resistance in female bearing pregnancies[26] and others reported similar findings to those for this cohort[10,13,15].

### Biology

The mechanisms by which sexual dimorphisms in PTB, PIHD, and GDM occur are unclear. Pregnancy is a physiological challenge for both mother and fetus. By secreting a number of hormones and growth factors into the maternal circulation, the placenta largely orchestrates maternal adaptations to pregnancy that result in markedly enhanced function of all her organs including kidneys, liver and cardiovascular system. These adaptations enable provision of an optimal environment for the fetus to grow and develop. However, they can unmask women already at cardiovascular and metabolic risk and compromise their and their babies’ health in the short and long terms[27]. Clifton presented a summary of several sex-specific strategies by which the fetus copes with adversity *in utero*[28]. Male and female fetuses adapt differently to developmental stressors and sex steroids have a profound influence on the development and progression of developmentally programmed disease states[1,28]. It is thought that the placenta plays an important role in obstetric complications, including PTB[28–30], PIHD[28,29,31] and GDM[28,32]. Placental gene expression in normal male versus female bearing pregnancies at term is significantly different[33] and may also be different in those with a pregnancy complication.

#### Preterm birth

Defective trophoblast invasion and colonization of the uterine spiral arterioles has been associated with a number of obstetrical complications, including PTB[29]. Incomplete remodeling of the spiral arterioles, a lesion classically associated with preeclampisa and IUGR, is also present in many cases of PTB[29]. Placentation is controlled, in part, by maternal-fetal immune interactions[34] and normal pregnancy is known to be an inflammatory state. In preterm birth before 32 weeks, male fetal sex has been associated with an increase in placental lesions, suggestive of a more aggressive maternal immune response against the invading interstitial trophoblast[30]. The interstitial trophoblast and maternal decidua are the most affected tissues, suggesting a local inflammatory response induced by the maternal immune system[35]. This aggressive maternal immune response could explain the male excess in spontaneous PTB but the exact mechanisms are unknown.

#### Pregnancy Induced Hypertensive Disorders

Defective placental invasion has also been associated with preeclampsia[29,34]. In general, in preeclampsia, remodeling of the utero-placental spiral arterioles fails mainly in the myometrial segments. In preeclampsia with intrauterine growth restriction the failure of myometrial segment remodeling is often associated with occlusive arterial lesions[29].

The “cross-over” in the sexual dimorphism for PIHD by length of gestation we have observed in our study was different for each analytic approach. According to the FAR approach, fetal sex was not a risk factor for PIHD resulting in birth between 30 and 36 weeks of gestation unlike observed sex-dependent early-onset and late-onset PIHD.

Most previous placenta studies have failed to consider sex bias in placental differentiation and function. Sex biases include different expression of genes, proteins and steroid pathways in response to an adverse maternal environment, including maternal asthma and preeclampsia[28]. In an adverse maternal environment the male fetus maintains his growth trajectory placing him at risk of PTB or stillbirth if an additional insult occurs. The female fetus adapts to an adverse maternal environment by slowing her growth trajectory. This allows her to survive if an additional insult occurs[28]. Also, since induction of labour is the only remedy for severe preeclampsia, the female predominance of iatrogenic PTB suggests that female fetuses are somehow able to induce hypertension in their mothers who suffer more severe PIHD than women carrying male fetuses. In response to adversity, the female feto-placental-unit may be able to increase maternal blood pressure and thereby improve placental perfusion pressure with the attendant maternal risk of PIHD. However, we are yet to understand how the female feto-placental unit can raise maternal blood pressure in mid gestation relatively more than that of the male.

We have recently shown in a meta-analysis of studies of genome-wide placental gene expression that there are 142 genes that are differentially expressed between placentas from male and female bearing non-pathological term pregnancies, with 75 genes more highly expressed in female placentas and 67 genes more highly expressed in male placentas. Genes were autosomal (60%), X-linked, as well as some Y chromosome genes[33]. The sex-specific differences in the placental transcriptome may explain sex differences in placental function, length of gestation and pregnancy outcomes.

An alternative explanation for the female excess in PIHD associated with PTB is a theory based on the influence of human chorionic gonadotropin (hCG). Circulating hCG is more abundant in pregnancies with preeclampsia[8], and high concentrations in the second and third trimesters have been associated with an increased risk for preeclampsia[36]. Interestingly, high concentrations of free β-hCG in the mother have also been associated with female fetal sex[37]. Furthermore, our meta-analysis of placental microarray data found that the *CGB* cluster of genes that encode β-hCG, are also more highly expressed in female placentas than in male placentas in uncomplicated pregnancies[33].

#### Gestational Diabetes Mellitus

Differences in the levels of steroid hormones in male and female fetuses may be expected to alter placental and fetal gene expression. This could also contribute to sex differences in fetal growth. Interestingly, sex differences in gene expression, glucose uptake, and metabolism are already apparent in embryos from as early as the 8-cell to blastocyst stages, long before the gonads differentiate[38]. At birth, cord arterial blood glucose levels are significantly higher in males than in females[21]. This is consistent with the view that male fetuses grow more rapidly and invest as little as possible in placental growth, while female fetuses invest in placental growth to protect them from a potentially poor maternal environment. The male growth strategy places males at greater risk in adversity[28,32]. Although insulin resistance is a feature of normal pregnancy[39], the male placenta may cause the mother to become relatively more insulin resistant and in association with other factors, this may increase the risk of GDM and macrosomia in male bearing pregnancies. However, if this is the case it is yet to be determined.

Despite the compelling findings of the current study, much remains unclear regarding the impact of biological sex on mechanisms underlying developmentally programmed responses[1].

### The Different Approaches to Analysis

The ambiguity in the results from other studies of sexual dimorphism of pregnancy outcomes compared to this study is mostly due to methodological differences, which this study aimed to address by using both traditional and FAR approaches.

The traditional approach is considered to be a descriptive approach towards perinatal outcome, since it assesses the relative difference in proportion of a perinatal event at a certain gestation compared to all the births at that gestation[24]. A disadvantage of the traditional approach is that it does not take into account that it is also the unborn fetuses that are at risk for the pregnancy outcome at a particular time in gestation. The FAR approach identifies fetuses as the candidates at risk for perinatal events. Therefore, it is thought that the FAR approach gives more insight into the causal links between length of gestation and perinatal outcomes[24].

In our opinion, both approaches are useful for perinatal research but it has to be clear that they measure different things. The traditional approach is suited for setting prognosis from early gestation and provides population prevalence, while the FAR approach provides a causal framework and the basis for obstetric intervention.

### Strengths and limitations

With 574,359 analysed births, this study is one of the largest to date on sexual dimorphism in pregnancy outcomes. The major strength of this type of population-based study lies in the unbiased statistical power that it provides.

As this was a retrospective design, we could only use variables that were included in the dataset. PIHD included both gestational hypertension and preeclampsia, as data on proteinuria were not collected by the SAPSC. It is however reasonable to assume that PIHD leading to (mostly iatrogenic) PTB would reflect preeclampsia. That is, the observed female predominance in PIHD with PTB likely reflects female predominance in preterm preeclampsia associated with labour induction.

Routine glucose challenge testing was introduced in South Australia in the early 1980s. GDM was uniformly reported from 1987 and therefore we were able to report data for a significant length of time. We are of course aware that there have been recent changes to the criteria for diagnosis of GDM as recommended by WHO that will increase the number of women diagnosed[40].

The SAPSC utilises notifications of births in South Australia made by hospital and homebirth midwives and hospital neonatal nurses on the SBR. The SBRs are checked manually for completeness and data discrepancies and go through a series of automated validation procedures during data entry. Validation studies by the SAPSC have shown that notifications of all births in South Australia on the SBR were robust for the parameters studied[41]. The subjects analysed in this study could therefore be considered as a true representation of the South Australian and Australian population.

### Conclusion and Future Directions

This large population based dataset covering 30 years of births in South Australia confirms the presence of marked sexual dimorphisms for PTB, PIHD, and GDM. We report that women carrying a male fetus are at increased risk for all PTB, spontaneous PTB, overall PIHD and GDM. Women carrying a female fetus are more at risk for PIHD complicated with PTB. Most interestingly, male fetuses show a 27% increased risk for extreme early PTB (20–24 weeks). Female fetuses have a 22% increased risk for PIHD complicated by PTB (25–29 weeks). In a clinical setting, fetal sex is important in determining the obstetric risks for pregnant women and perinatal outcomes for infants.

This study suggests that fetal sex should be taken into account in further studies on obstetric complications and their mechanisms. Further research on sex differences in placental function and maternal adaptation to pregnancy is required to delineate the causal molecular mechanisms in sex-specific pregnancy outcome. Identifying these mechanisms may inform fetal sex specific tailored antenatal care and infant care in the neonatal nursery and on into childhood.
